# Landscape of Global Oncology Research and Training at National Cancer Institute–Designated Cancer Centers: Results of the 2018 to 2019 Global Oncology Survey

**DOI:** 10.1200/JGO.19.00308

**Published:** 2019-11-22

**Authors:** Rachel M. Abudu, Mishka K. Cira, Doug H.M. Pyle, Kalina Duncan

**Affiliations:** ^1^ Frederick National Laboratory for Cancer Research, Frederick, MD; ^2^ ASCO, Alexandria, VA; ^3^ National Cancer Institute Center for Global Health, Rockville, MD

## Abstract

**PURPOSE:**

The National Cancer Institute (NCI)–Designated Cancer Centers (NDCCs) are active in global oncology research and training, leading collaborations to support global cancer control. To better understand global oncology activities led by NDCCs, the NCI Center for Global Health collaborated with ASCO to conduct the 2018/2019 NCI/ASCO Global Oncology Survey of NDCCs.

**METHODS:**

Seventy NDCCs received a two-part survey that focused on global oncology programs at NDCCs and non–National Institutes of Health (NIH)-funded global oncology projects with an international collaborator led by the NDCCs. Sixty-seven NDCCs responded to the survey. Data were coded and analyzed by NCI-Center for Global Health staff.

**RESULTS:**

Thirty-three NDCCs (47%) reported having a global oncology program, and 61 (87%) reported a collective total of 613 non–NIH-funded global oncology projects. Of the NDCCs with global oncology programs, 17 reported that trainees completed rotations outside the United States and the same number enrolled trainees from low- and middle-income countries (LMIC). Primary focus areas of non–NIH-funded projects were research (469 [76.5%]) and capacity building or training (197 [32.1%]). Projects included collaborators from 110 countries; 68 of these were LMIC.

**CONCLUSION:**

This survey shows that there is a substantial amount of global oncology research and training conducted by NDCCs and that much of this is happening in LMIC. Trends in these data reflect those in recent literature: The field of global oncology is growing, advancing scientific knowledge, contributing to building research and training capacity in LMIC, and becoming a recognized career path. Results of the 2018 Global Oncology Survey can be used to foster opportunities for NDCCs to work collaboratively on activities and to share their findings with relevant stakeholders in their LMIC collaborator countries.

## INTRODUCTION

Mortality from cancer is on the rise, and by 2020 it is estimated that almost 70% of cancer cases will be in low- and middle-income countries (LMIC).^1^ Global oncology research plays a crucial role in understanding causes and patterns of rare cancers,^1,2^ assessing novel low-cost therapies,^3,4^ addressing cancer health disparities,^5^ and studying how interventions can best be implemented in local communities.^6-8^ Much of this groundbreaking work occurs through research and training collaborations between institutions in high-income countries (HIC) and LMIC. These collaborations aim to benefit both HIC and LMIC by building cancer research capacity, sharing and building research resources and infrastructures that leverage data and unique experiences of country partners, answering questions about cancers and cancer pathways otherwise under-represented in global research, driving breakthroughs, and improving health outcomes.^2,5,9,10-14^

The stated mission of the US National Cancer Institute (NCI) is that it leads, conducts, and supports cancer research across the nation to advance scientific knowledge and help all people live longer, healthier lives.^15^ As part of this mission, the NCI supports 71 NCI-Designated Cancer Centers (NDCCs) tasked with delivering state-of-the-art cancer research and care.^10,16^ The NCI’s extramural research portfolio includes awards to domestic (US) institutions, grants awarded to foreign institutions, and grants with a US-based institution and one or more foreign collaborators. In fiscal year 2018 (FY2018), grants awarded to a foreign institution and grants with one or more foreign collaborators made up approximately 11.0% of the overall NCI FY2018 extramural portfolio.^17^ The majority (76.3%) of FY2018 grants with foreign collaborators were led by NDCCs or their affiliated academic institutions.^17^

**CONTEXT**

**Key Objective**
Understand the global oncology program offerings and projects at National Cancer Institute–Designated Cancer Centers (NDCCs) in 2018 and 2019.
**Knowledge Generated**
Thirty-three NDCCs (47%) reported having a global oncology program at their center, and 61 NDCCs (87%) reported a collective total of 613 non–National Institutes of Health (NIH)-funded global oncology projects. Primary focus areas of non–NIH-funded projects were research (469 projects [76.5%]) and capacity building or training (197 projects [32.1%]), and projects included collaborators from 110 countries.
**Relevance**
Benchmarking global oncology programs and non–NIH-funded global oncology projects at NDCCs provides a more holistic view of cancer centers’ commitment to global oncology and offers insights on how to grow the field of global oncology.

Information on global oncology activities are publicly available from databases such as the National Institutes of Health (NIH) World RePORT^18^ (https://worldreport.nih.gov), which captures the NCI-funded extramural research portfolio with foreign collaborations, the International Cancer Research Partnership database^19^ (www.icrpartnership.org), and The GO (Global Oncology) Map^20^ (www.thegomap.org). However, there is limited literature detailing the full scope of global oncology research and training collaborations that are conducted specifically by NDCCs.^21^ To address this gap, the NCI Center for Global Health (CGH) fielded surveys to NDCCs in 2012,^22^ 2014,^23^ and 2018 to 2019^24^ to better understand the landscape of non–NIH-funded global oncology activities and to augment known information on global oncology investments. This analysis focuses on the 2018 to 2019 survey results.

NCI/CGH collaborated with ASCO to develop a survey module on global oncology programs, representing the first time that NDCCs have been asked by NCI in a standardized method about the existence and offerings of the global oncology programs at their cancer centers. Questionnaire design for this module was done in coordination with members of ASCO’s Academic Global Oncology Task Force, an ASCO Board-appointed body of ASCO members with expertise in global oncology convened to make recommendations on ways that ASCO can support global oncology as an academic discipline. The 2018 to 2019 survey had three key objectives: (1) describe the non–NIH-funded global oncology activities led by NDCCs in a centralized resource, (2) identify areas where NDCCs are working to promote research partnerships and potential areas for increased collaboration, and (3) use the results to convene NDCCs focused on global oncology opportunities.

## METHODS

### Survey Design and Distribution

The survey consisted of two sections: (1) 23 questions focused on global oncology programs at the NDCCs (ie, programs that include faculty engagement and training opportunities in global oncology), and (2) 27 questions focused on non–NIH-funded global oncology projects (ie, discrete research or capacity-building/training projects with international collaborators) led by NDCCs. Responses from NDCCs could be submitted via an online Google Forms survey or via an Excel spreadsheet that mirrored the fields in the online survey. The survey tools are available in the full report.^24^

The survey was piloted to seven NDCCs in early 2018, and minor adjustments were subsequently made to clarify intent of some survey questions. The revised survey was sent to all NDCCs between March and May 2018, with an initial survey closing date of October 2018. For each NDCC, the survey was sent to the NDCC Director, Administrator, and any relevant global oncology contact persons identified by the NDCC leadership, the NCI Office of Cancer Centers, ASCO, and NCI/CGH staff.

### Project Coding

NDCCs were asked to code projects to one or more project types (eg, research or capacity building), Common Scientific Outline (CSO) codes, cancer sites (eg, breast, prostate, and so on), and project funding source(s). Three NCI/CGH staff reviewed these codes for agreement with the project title and abstract and, where appropriate, applied additional codes to further capture themes that arose in the data set. Two NCI/CGH staff reviewers coded every project to the new codes, and a third NCI/CGH reviewer reconciled any discrepancies.

### NCI FY2018 Extramural Research Portfolio

The NCI-funded FY2018 international extramural research portfolio, evaluated by authors R.M.A. and K.D. at NCI/CGH, has been used as a comparison data set to the 2018 to 2019 Global Oncology Survey data presented in this article. This portfolio was prepared using NIH’s internal IMPAC II (Information for Management, Planning, Analysis, and Coordination) data system and includes any extramural grant funded by NCI during FY2018 with an international collaborator.^17^ NCI projects are coded to CSO and cancer site codes.

### Statistical Analysis and Review

Data were analyzed in Microsoft Excel and in Statistical Analysis Software (SAS). In March 2019, a draft version of the report was circulated to 70 NDCCs for review (at the time of the survey there were 70 NDCCs), and additional data revisions were included in the final report.

## RESULTS

A total of 67 NDCCs (96% response rate) responded to the survey (Table 1).

**TABLE 1:**
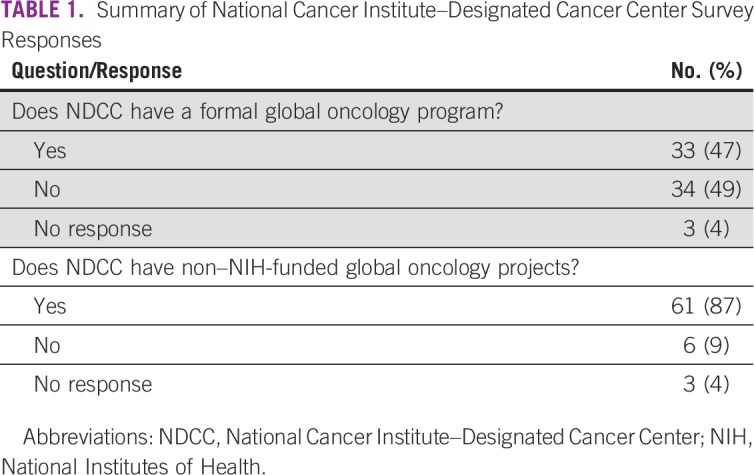
Summary of National Cancer Institute–Designated Cancer Center Survey Responses

### Global Oncology Programs

Thirty-three of 70 NDCCs (47%) reported having a global oncology program at their institution, and 27 reported having a designated program lead. The data in this section represent the 33 NDCCs that reported having a global oncology program. NDCCs could self-define as a global oncology program and, as a result, observed program structures varied from more formalized academic programs to informal groups of interested investigators who conduct global activities. Top program focus areas included research (24 NDCCs), capacity building and training (15 NDCCs), and LMIC collaboration (15 NDCCs; Table 2). Codes were on the basis of program descriptions supplied by NDCCs, and programs could receive more than one code. Thirteen NDCCs reported having one to 10 faculty members engaged in global oncology, and 19 NDCCs reported having 11 or more faculty members engaged in global oncology. Twenty-two NDCCs reported receiving both external funding support and institutional funding for global oncology activities. Five NDCCs reported receiving only external funding, and three NDCCs reported receiving only institutional funding support for global oncology activities.

**TABLE 2:**
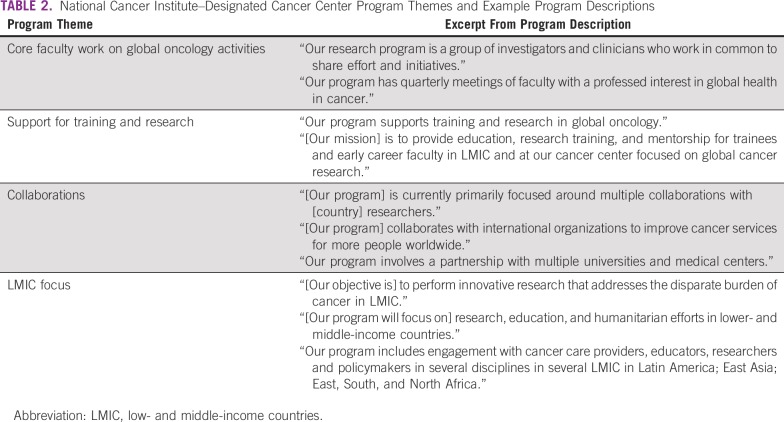
National Cancer Institute–Designated Cancer Center Program Themes and Example Program Descriptions

Twenty-seven NDCCs reported providing an average of 1 to 2 days of global oncology training to trainees. NDCCs reported that trainees (in specialties ranging from medical oncology to radiology) participated in global oncology training and had an interest in pursuing global oncology as a part of their future careers. NDCCs also reported offering training opportunities to students from LMIC, either through enrolled opportunities (ie, LMIC students were enrolled in their programs [17 NDCCs]) or nonenrolled opportunities (ie, LMIC students participated in capacity-building activities or clinical observerships [15 NDCCs]).

### Non–NIH-Funded Global Oncology Projects

A total of 61 NDCCs (87%) reported having one or more non–NIH-funded global oncology projects, and 613 non–NIH-funded projects were reported overall.

**FIG 1:**
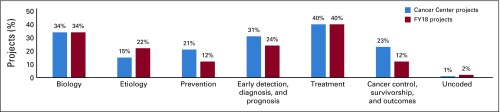
Non–National Institutes of Health-funded National Cancer Institute–Designated Cancer Center global oncology projects by Common Scientific Outline code compared with fiscal year (FY) 2018 National Cancer Institute–funded international grants.

The number of projects reported per NDCC ranged from one to 58, with 20 NDCCs reporting 10 or more projects. Top project focus areas were research (77%) and capacity building or training (32%). Secondary topics included cancer screening (8.8%), clinical trials (6.9%), implementation (6.7%), networks or working groups (5.4%), cancer registries (3.9%), and pathology (3.4%; Table 3). Projects could be coded to more than one primary or secondary focus area. Top CSO codes covered by NDCC global oncology projects included treatment (40%); biology (34%); and early detection, diagnosis, and prognosis (31%; Fig 1). The top three cancer sites reported by NDCCs following non–site-specific cancers (32%) were breast (18%), cervix (7%), and stomach or GI (5%).

**TABLE 3:**
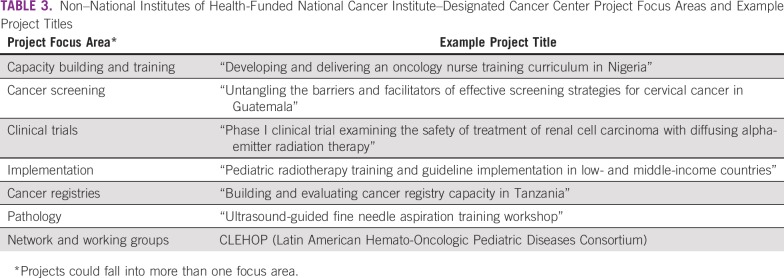
Non–National Institutes of Health-Funded National Cancer Institute–Designated Cancer Center Project Focus Areas and Example Project Titles

**FIG 2:**
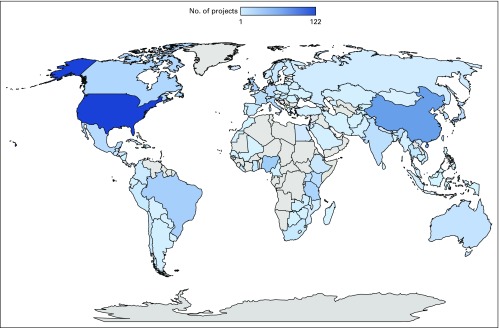
Map of non–National Institutes of Health-funded National Cancer Institute–Designated Cancer Center global oncology projects by collaborator country.

**FIG 3:**
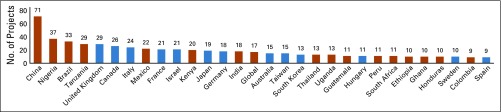
Non–National Institutes of Health-funded National Cancer Institute–Designated Cancer Center global oncology projects by collaborator country. Low- and middle-income countries are shown in orange, and figure includes projects defined as global.

Non–NIH-funded global oncology projects included collaborators from 110 countries spanning seven world regions (East Asia and Pacific: 154 projects, 35 NDCCs; Europe and Central Asia: 131 projects, 39 NDCCs; Latin America and the Caribbean: 102 projects, 33 NDCCs; Middle East and North Africa: 41 projects, 21 NDCCs; North America: 139 projects, 44 NDCCs; South Asia: 28 projects, 15 NDCCs; and Sub-Saharan Africa: 142 projects, 34 NDCCs; Fig 2). Collaborators came from 42 HIC and 68 LMIC, 16 of which were low-income countries. Sixteen of the top 30 collaborator countries (ranked by number of non–NIH-funded projects) were LMIC (Fig 3).

NDCCs reported having more than 150 different funders for their non–NIH-funded global oncology projects. Examples include institutional funds (within the NDCC, 252 projects), nonprofit organizations (133 projects), industry (33 projects), and other funding sources including US government agencies (non-NIH) and international sources (84 projects). Funding sources were not provided for 105 projects, and 34 projects were listed as unfunded.

A full summary report of the 2018 to 2019 Global Oncology Survey was developed by NCI/CGH and is available on the NCI Web site.

## DISCUSSION

Results of the 2018 to 2019 Global Oncology Survey of NDCCs describe the scope of global oncology programs and activities of NDCCs and highlight NDCCs’ commitment to global oncology beyond NIH-funded grants. Trends in these data reflect those in recent literature: The field of global oncology is growing,^25^ advancing scientific knowledge,^26^ contributing to building research and training capacity in LMIC,^27,28^ and becoming a desired career path.^29^

### A Global Oncology Community

Since the 2012 survey, the number of NDCCs reporting global oncology activities has grown substantially. Fifty-five of the (at that time) 68 NDCCs (80.9%) reported global oncology activities in 2012,^22^ and in the 2018 to 2019 survey, 65 of 70 NDCCs (92.9%) reported global oncology activities. Previous surveys did not capture details at the project level, so it is not possible to compare total projects reported over time. However, in the 2018 to 2019 survey, 61 NDCCs reported a total of 613 non–NIH-funded projects. Taken together with the approximately 560 extramural grants with a foreign collaborator led by NDCCs in the NCI FY2018 extramural research portfolio,^17^ it is clear that NDCCs are engaging in substantial global oncology activity. Growth in the number of NDCCs reporting global oncology activities is mirrored by the increasing number of global cancer research publications worldwide.^25^

Nearly half of all NDCCs report having a global oncology program. There may be additional NDCCs with faculty conducting global oncology research, despite not having a program. Although this information was not captured in previous surveys, this serves as baseline information for future tracking of growth in the field of global oncology at NDCCs.

The survey identified diverse funding sources for global oncology activities, including external grants, institutional funding, and funding from other sectors. The diversity of funding sources shows that a variety of actors are interested in supporting global cancer research and training. It also indicates that no single funder can sustain the growing interest in research in this field, as reflected elsewhere in the literature.^10^

### Advancing Scientific Knowledge of Cancer

NDCC global activities reflect common global oncology scientific priority recommendations outlined in the literature: prioritize research across all areas of the cancer continuum^5,9,30-32^ and prioritize research in high-burden cancers.^9,30,31,33,34^ NDCC projects were more likely than NIH-funded FY2018 grants with foreign collaborators to be coded to cancer control, survivorship, and outcomes research (CSO code #6).^17^ Within CSO code #6, one of the identified global priorities is the need for prioritization of implementation research.^8,35^ Although the initial analysis identified that almost 7% of non–NIH-funded projects had a secondary focus on implementation research, further analysis is needed to fully understand how NDCCs are addressing these stated priority areas.

Most non–NIH-funded global oncology projects from NDCCs that were cancer site specific addressed breast, cervical, and stomach or GI cancers. In addition, more than one-third of NDCCs reported projects that included focus on childhood cancers. Focus on these cancers aligns with Global Burden of Disease data^36^ and may indicate alignment of research focus with disease burden.

### Contributing to Building Research and Training Capacity in HIC and LMIC

Collaboration to build research and training capacity, particularly within LMIC, was a theme seen across NDCC global oncology activities. Non–NIH-funded global oncology projects from NDCCs were nearly twice as likely as NCI-funded FY2018 projects to include collaborators from LMIC (a total of 68 LMIC).^17^ Almost one-third focused on capacity building or training, ranging from research infrastructure to human resource and skill training. Of the 33 NDCCs reporting a global oncology program, the majority reported providing at least some training in global oncology to their trainees, more than half reported enrolling trainees from LMICs, and almost half reported providing capacity-building opportunities for LMIC counterparts. Building capacity in LMICs to conduct research strengthens local capacity, provides increased global research opportunities, and supports the global cancer research community to advance scientific knowledge of cancers in all communities.^5,27,28^

### Global Oncology Is a Desired Career Path at NDCCs

Importantly, this survey shows that global oncology is a desired career path at NDCCs. For NDCCs that reported having a global oncology program, the majority reported having 11 or more faculty engaged in their global oncology program. This number does not capture faculty involvement at NDCCs without a global oncology program (see Limitations section). NDCC trainees who participated in global oncology training also reported having an interest in pursuing global oncology as part of their career. Notably, NDCCs that did not have global oncology training related to pediatric oncology reported an interest among their trainees in receiving such training in the future. As NDCCs continue to strengthen training in global oncology, there is an opportunity to develop increased training and career pathways for specialty trainees. Building the global oncology workforce is critical to addressing clinician shortages and to expanding cancer research across diverse populations.^28,37,38^ Academic careers in global health have unique challenges, such as limited opportunities for global health research funding, limited academic advancement, and challenges balancing research demands abroad with personal lives.^39^ Developing new ideas to build out global oncology as an academic career path will be important for creating a pipeline for future global oncology researchers and clinicians.^28,29,40,41^ As these career paths develop at NDCCs, it will be important to consider how to craft these programs ethically and equitably so that they avoid the pitfalls of parachute research and global health consulting malpractice.^42,43^ The findings of the ASCO Academic Global Oncology Task Force (which include recommendations in the areas of global oncology training and mentorship, research, and practice) are expected to be disseminated in a forthcoming publication.

### Strengthening Information Sharing Within the Global Oncology Community

The 2018 to 2019 Global Oncology Survey of NDCCs is intended to serve as a centralized resource for NDCCs and their partners to examine the breadth of global activities across the NDCC community and to identify potential collaborations. NDCCs reported some challenges in amalgamating global oncology data (further articulated in the Limitations section); as such, it may be helpful to develop community-wide standards for collecting data and reporting on global oncology programs and projects. This will assist in more accurate overall reporting and building the case for supporting NDCC-led global oncology activities.

This report is intended to be a catalyst and convening tool for strategic discussions among NDCCs. In addition, the report can inform the efforts of other stakeholders, such as ASCO and other oncology societies, to support the profession, and their members who are interested in this field. One opportunity will be at the 8th Global Cancer Research Symposium, a scientific satellite meeting at the 2020 Annual Consortium of Universities for Global Health Conference to be held on April 17, 2020, in Washington, DC. The Symposium aims to convene individuals working in global oncology to discuss trends, gaps, and potential collaborations in global cancer research and control.

### Limitations

Factors affecting data accuracy and completeness of this survey are as follows: (1) data were self-reported by NDCCs, and data completeness was at the discretion of responding institutions; (2) data may vary depending on who within the NDCC supplied the information; (3) in some cases, these data were not held centrally and were difficult for respondents to amalgamate; (4) NDCCs varied in the types of projects they reported (eg, some NDCCs reported unfunded work whereas others did not); (5) projects were self-reported, so it is possible that multiple NDCCs working on the same or a similar project reported these projects individually; and (6) the terms “global oncology” and “global oncology programs” were not defined in the survey because a current widely accepted definition of global oncology does not yet exist.^32^ Therefore, NDCCs may have had different understandings of the definition of “global oncology program,” which may have affected the response rate. As a result, data on faculty and trainee engagement in global oncology activities, and the total number of non–NIH-funded projects, may be under- or over-reported.

To confirm accuracy of reporting on non–NIH-funded projects, a subset of projects (61 projects [10%]) was scanned for potential duplicates within the NIH-funded portfolio. Of the 61 projects reviewed, two (3.3%) had received funding from NIH, 55 (89.7%) had not received funding from NIH, and funding status for four projects (6.5%) could not be determined. This subanalysis provides a high level of confidence that most reported projects are not represented in the NIH portfolio.

In conclusion, the 2018 to 2019 Global Oncology Survey of NDCCs demonstrates a robust level of global oncology activities occurring at NDCCs and is the first of its kind to provide a baseline measurement of the number and types of NDCC global oncology programs. Understanding the broad reach of global oncology programs and non–NIH-funded projects that are ongoing at NDCCs is important for facilitating collaborations between NDCCS. This report should serve as a catalyst for strategic discussions on coordination and investment. The NDCC global oncology community is growing and has a unique opportunity to advance scientific knowledge, build research capacity in HIC and LMIC, and strengthen the global oncology academic career path at NDCCs. Future iterations of the survey have the potential to capture greater detail on global oncology offerings at NDCCs and align with ongoing priorities of the NDCC global oncology community.
